# COVID-19 vaccination schedule, associated factors, and reasons for refusal in adults and the elderly: a population-based cross-sectional study, Rio Grande, Brazil, 2023

**DOI:** 10.1590/S2237-96222026v35e20250503.en

**Published:** 2026-03-10

**Authors:** Aline Augusta Medeiros Rutz, Mirelle de Oliveira Saes, Rosália Garcia Neves, Thales Rodrigues Almeida, Renata Cristina da Silva Martins, Suele Manjourany Silva Duro

**Affiliations:** 1 Universidade Federal de Pelotas, Programa de Pós-Graduação em Enfermagem, Pelotas, RS, Brazil Universidade Federal de Pelotas Programa de Pós-Graduação em Enfermagem Pelotas RS Brazil; 2 Universidade Federal do Rio Grande, Programa de Pós-Graduação em Ciências da Saúde, Rio Grande, RS, Brazil Universidade Federal do Rio Grande Programa de Pós-Graduação em Ciências da Saúde Rio Grande RS Brazil

**Keywords:** Vaccination Coverage, Immunization Schedule, Vaccination, COVID-19, Cross-Sectional Studies, Cobertura de Vacunación, Esquemas de Inmunización, Vacunación, COVID-19, Estudios Transversales

## Abstract

**Objective:**

To estimate the prevalence of COVID-19 vaccination schedule completion and to investigate associated factors and reasons for refusal among adults and elderly residents of Rio Grande, Rio Grande do Sul state, Brazil.

**Methods:**

This was a cross-sectional study with participants from the Adult and Elderly post-COVID-19 Infection Health Indicator Follow-up, conducted in 2022-2023. The outcome was the COVID-19 vaccination schedule. Having had at least three doses of the vaccine was considered to be complete vaccination. The exposure variables were sociodemographic and morbidity characteristics. Reasons for not completing the vaccination schedule were also investigated. Prevalence rates and unadjusted and adjusted prevalence ratios were calculated.

**Results:**

Of the 1,925 respondents, 83.3% received all three doses of the vaccine. Vaccination prevalence was higher among women (85.5%), older adults (92.6%), respondents who had never smoked (83.3%) or were former smokers (87.6%), those with multimorbidity (88.6%), and those who had been informed about the importance of the vaccine (85.1%). Among those who did not complete the vaccination schedule, 15.8% did not do so because they had a reaction to the vaccine; the vaccine was not available for 8.5% when they went to get vaccinated and they did not return; 8.1% knew someone who had a reaction and were apprehensive; and 7.3% stated they had already had COVID-19.

**Conclusion:**

In all, 83.3% completed the vaccination schedule of at least three COVID-19 vaccine doses. Adherence to vaccination should be encouraged, especially in groups with lower coverage rates, such as men, younger people, and those without comorbidities.

## Resumen

**Objetivo:**

Estimar la prevalencia de la realización del esquema de vacunación contra COVID-19 e investigar los factores asociados y las razones de rechazo entre adultos y ancianos residentes de Rio Grande, estado de Rio Grande do Sul, Brasil.

**Métodos:**

Estudio transversal con participantes del Monitoreo de Indicadores de Salud en Adultos y Ancianos tras la Infección por COVID-19, realizado entre 2022 y 2023. El resultado fue el esquema de vacunación contra la COVID-19. Se consideró la recepción de al menos tres dosis de la vacuna. Las variables de exposición fueron las características sociodemográficas y de morbilidades. También se investigaron las razones para no completar el esquema de vacunación. Se calcularon las prevalencias, así como las razones de prevalencia brutas y ajustadas.

**Resultados:**

De los 1925 encuestados, el 83,3 % recibió las tres dosis de la vacuna. La prevalencia de vacunación fue mayor entre las mujeres (85,5%), los adultos mayores (92,6%), los encuestados que nunca habían fumado (83,3%) o eran exfumado­res (87,6%), aquellos con multimorbilidad (88,6%) y aquellos que habían sido informados sobre la importancia de la vacuna (85,1%). De quienes no completaron el esquema de vacunación, el 15,8% no lo hizo por presentar una reacción; el 8,5% no contaba con el inmunobiológico al momento de vacunarse y no volvió a solicitarlo; el 8,1% conocía a alguien que presentó una reacción y se mostró aprensivo; y el 7,3% afirmó haber tenido COVID-19.

**Conclusión:**

El 83,3% completó el esquema de vacunación de al menos tres dosis contra la COVID-19. Se debe fomentar la adherencia a la vacunación, especialmente en grupos con tasas de cobertura más bajas, como hombres, jóvenes y personas sin comorbilidades.

**Ethical aspects**

This research respected ethical principles, having obtained the following approval data:

Research ethics committee: Universidade Federal do Rio Grande

Opinion number: 4,375,697

Approval date: 3/11/2020

Certificate of submission for ethical appraisal: 39081120.0.0000.5324

Informed consent record: Ethical principles were ensured through the reading of the Informed Consent record, respecting the right not to participate in the research and the anonymity of the subjects.

## Introduction 

COVID-19 was identified in December 2019 in China. Due to its high transmissibility, the World Health Organization (WHO) declared it to be a public health emergency of international concern, advising countries to adopt preventive measures such as social isolation and use of face masks. Vaccines have emerged as an important tool for the prevention of this communicable disease ^1^^,^^2^.

In May 2023, the WHO Director-General declared the end of the COVID-19 public health emergency. Despite following the recommendation of the WHO Emergency Committee, the Director warned that COVID-19 continued to be a threat to global health. Therefore, countries, their health systems and their populations should continue to adopt preventive measures. Vaccination stood out among the main recommendations ^3^.

In November 2022, the Brazilian Ministry of Health emphasized that Brazilians needed to receive booster doses against coronavirus to ensure effective protection. However, that same year, more than 80 million people failed to go to vaccination centers to receive their first booster dose. The strategy of reinforcing the vaccination schedule increased protection against severe COVID-19 cases and deaths more than fivefold. In Brazil, by September 2023, only 16% of the population had been immunized with the bivalent vaccine ^4^^,^^5^^,^^6^.

There are significant disparities in vaccination rates across different countries, ranging from 5% in Haiti to 100% in the United Arab Emirates ^6^. Standing out among the reasons for refusal are fear of side effects, mistrust of public policies, origin of vaccines, disbelief in their efficacy and lack of vaccine safety. Other notable reasons include: having already been infected with the virus, lack of clinical data on the vaccine, believing that COVID-19 is a harmless disease, believing in conspiracy theories, and not being in a high-risk group for severe COVID-19 illness ^7^^,^^8^.

This study is justified by the importance of identifying adherence to COVID-19 vaccination, profiling those who have been least vaccinated. It is believed that, in this way, public policies can be proposed and developed aiming to expand vaccination coverage in the population.

The objective of this study was to estimate the prevalence of adherence to the COVID-19 vaccination schedule and to investigate associated factors and reasons for refusal among adults and elderly residents of Rio Grande, a municipality located in the Southern region of Brazil.

## Methods 

Design

This is a cross-sectional study nested within a cohort. The study used data from the second data collection of the longitudinal study entitled "Post-COVID-19 Infection Follow-up of Health Indicators of Adults and Elderly living in Rio Grande, Rio Grande do Sul State".

Participants and setting

The research was conducted in Rio Grande, Rio Grande do Sul state. Located in the far south of the state, the municipality has an estimated population of 210,000 inhabitants. As of January 2024, it had approximately 60,834 confirmed COVID-19 cases with a mortality rate of approximately 346.4 per 100,000 inhabitants ^9^.

In the baseline study, the sample included individuals aged 18 years or older who had a confirmed diagnosis of COVID-19 via Reverse Transcription Polymerase Chain Reaction (RT-PCR) between December 2020 and March 2021; who presented symptoms during the infection; and who lived in Rio Grande. Individuals with functional limitations and/or advanced neurological diseases who were not able to answer the questionnaire and those deprived of liberty (prisons or long-term care facilities for the elderly) were excluded. Data collection was carried out in two consecutive stages: 1) Telephone collection: Up to five telephone contacts were made with each individual eligible for the study. These contacts were made on alternate days and times. After five unsuccessful contacts, a standardized message was sent to the telephones that had WhatsApp. For individuals who responded to the message sent, a telephone or in-person interview was scheduled, according to the respondent's preference. Those who did not answer the telephone or respond to the message were allocated to the next stage, home data collection. 2) Home data collection: In this stage of the study, the identified interviewer went to the respondent's home to conduct the interview with those who were not located during the telephone data collection stage. The instrument and its administration method were the same as for telephone data collection. Individuals who could not be located after five telephone contact attempts, one via WhatsApp, and three home visits were considered to be losses.

In order to locate the sample, in the baseline study contact was made with the Rio Grande Municipal Health Department and a list of all reported COVID-19 cases was obtained. Symptomatic individuals with a positive RT-PCR test result and who had provided complete data (name, address, telephone and presence of symptoms) were selected. A total of 3,822 were eligible for the study; of these, 2,919 individuals were interviewed in this stage ^10^.

For the follow-up study, the 2,919 individuals interviewed at baseline were contacted. Data collection occurred in two stages: 1) telephone interviews and 2) home interviews, as was the case during the baseline study. It began with telephone calls conducted by previously trained interviewers. When possible, the interview was conducted on the first telephone contact, or, if necessary, a date and time were scheduled. For individuals who did not answer five calls, contact was attempted via WhatsApp. In addition to telephone interviews, home visits were conducted to collect data from those who were hesitant to answer the phone and from those who did not answer any of the five telephone attempts.

Data collection took place between December 2022 and May 2023. The instrument contained general data; questions about remaining COVID-19 infection symptoms; social support and receipt of guidance; lifestyle habits; behaviors; health; use of health services; vaccination; and socioeconomic data.

Variables

The study outcome was measured using the question "How many doses of the COVID-19 vaccine have you received?". This variable was categorized into 0-2 and 3-4 doses (maximum number of doses available at the time of data collection). Individuals who received at least three doses were considered to be vaccinated.

The independent variables used were: sex (male; female); age group in years (up to 29 years; 30-39; 40-49; 50-59; and 60 years or more); economic level, according to the Brazilian Criteria proposed by the Brazilian Association of Survey Companies (Associação Brasileira de Empresas de Pesquisa, ABEP) (A/B; C; D/E), where A is the wealthiest category and D/E the poorest ^11^; race/skin color (White; Black; mixed-race/Asian /Indigenous); marital status (with partner; without partner); smoking (no; yes; former smoker); multimorbidity (no; yes); and receiving guidance on the importance of vaccination (no; yes). For the race/skin color variable, data was collected in all five categories, but for statistical analysis purposes, mixed-race, Asian and Indigenous were combined into one. Multimorbidity was defined as when the individual had two or more concomitant problems from the following list: mental or emotional illness; respiratory problems; osteoporosis or joint problems; hypertension; diabetes; heart problems; eye problems; cancer; neurological diseases.

The following question was asked in order to assess the reasons for not completing the vaccination schedule: "Do you have a complete vaccination schedule in accordance with your age?" (no; yes). In this case, a complete vaccination schedule was considered to have been received when the individual had received at least three doses of the COVID-19 vaccine. If the answer was "no", the reason was then investigated as follows: "I will read some reasons and you can tell me which one(s) are closest to your reality". The reasons are: 1) I already had COVID-19; 2) I had a reaction and got scared; 3) fear/dislike of needles; 4) a relative/friend said it's harmful; 5) I know someone who had a reaction; 6) lack of professional guidance; 7) difficulty accessing the vaccine; 8) vaccines are useless; 9) one dose is enough; 10) didn't know/don't know when the campaign is; 11) when I went to get vaccinated it wasn't available and I didn't go back to get vaccinated; 12) I saw on TV or social media that the vaccine doesn't work or is harmful.

Statistical methods

Descriptive analysis was performed to obtain proportions, and bivariate analysis of the outcome was conducted according to independent variables using the chi-square test for heterogeneity and linear trend. Unadjusted and adjusted analyses were conducted using Poisson regression, with robust variance estimation, according to a hierarchical model. Variables with a p-value≤0.20 were retained in the model to control for possible confounding factors. The hierarchical model consisted of three levels distributed as follows: 1) sex, age, economic level, race/skin color and marital status; 2) smoking; 3) multimorbidity and receipt of guidance on the importance of vaccination against COVID-19. Data analysis was performed using Stata software, version 15.0 (Stata Corporation, College Station, Texas, USA), taking a 5% significance level.

## Results

A total of 1,925 individuals were interviewed in the follow-up study, representing 65.9% of the initial sample of 2,919 individuals. Of these, 1,179 were women (61.3%), 1,197 were under 49 years old (62.2%), 1,148 reported having a partner (59.9%), 1,486 self-identified as White (77.6%), and 1,499 had never smoked (78.9%). Additionally, 941 were in socioeconomic level C (54.1%), 855 had multimorbidity (46.3%), 1,153 received guidance on the importance of COVID-19 vaccination (60.4%), and 1,046 reported experiencing some side effect after vaccination (58.6%) (Table 1).

Prevalence of receiving three or more doses of the aforementioned vaccine was 83.3% (n=1,511), being statistically more frequent among women, older individuals, former smokers and those who have never smoked, people with multimorbidity, and those who received guidance on the importance of vaccination. No statistical differences were found in vaccination prevalence according to marital status, race/skin color or economic level (Table 1).

After adjusted analysis, it was shown that women were 7% more likely than men (PR 1.07; 95%CI 1.02; 1.12) to receive the vaccine. There was a direct relationship between the probability of receiving the vaccine and increasing age; among those aged 60 or older, it was 1.32 times higher than among individuals up to 29 years old (PR 1.32; 95%CI 1.22; 1.43). People with multimorbidity (PR 1.07; 95%CI 1.02; 1.11) and those who received guidance on the importance of vaccination (PR 1.06; 95%CI 1.02; 1.11) were also more likely to get vaccinated (Table 2).

Regarding side effects, the most frequently reported were body and/or joint pain (79.7%), headache (59.1%), fever (56.3%) and chills (42.0%) (Figure 1).

**Table 1:** Distribution of the sample and prevalence (%) of the COVID-19 vaccination schedule according to independent variables. Rio Grande, 2023 (n=1,925)

Variables	Sample (%)	Vaccination schedule (%)	p-value
Total sample		1,511 (83.3)	
Sex			0.001
Male	746 (38.8)	554 (79.6)	
Female	1,179 (61.3)	955 (85.5)	
Age group (years)			<0.001
Up to 29	311 (16.2)	207 (70.4)	
30-39	442 (23.0)	331 (78.4)	
40-49	444 (23.1)	352 (84.6)	
50-59	339 (17.6)	280 (88.9)	
60 or over	389 (20.2)	339 (92.6)	
Marital status			0.256
Has a partner	1,148 (59.9)	889 (82.5)	
Does not have a partner	770 (40.2)	616 (84.5)	
Race/skin color			0.160
White	1,486 (77.6)	1.172 (83.8)	
Black	198 (10.3)	158 (84.0)	
Mixed-race/Asian/Indigenous	230 (12.0)	169 (78.6)	
Economic level			0.538
A/B (wealthiest)	701 (40.3)	592 (84.7)	
C	941 (54.1)	779 (82.9)	
D/E (poorest)	98 (5.6)	80 (81.6)	
Tobacco smoking			0.033
No	1,499 (78.9)	1.185 (83.3)	
Yes	166 (8.7)	124 (77.5)	
Former smoker	236 (12.4)	197 (87.6)	
Multimorbidity			<0.001
No	992 (53.7)	748 (78.7)	
Yes	855 (46.3)	733 (88.6)	
Guidance on importance of vaccination			0.009
No	756 (39.6)	575 (80.4)	
Yes	1,153 (60.4)	931 (85.1)	
Doses of COVID-19 vaccine			
0-2 doses	304 (16.8)	-	
3 or 4 doses	1,511 (83.3)	-	
Post-vaccination side effects			
No	739 (41.4)	-	
Yes	1,046 (58.6)	-	

**Table 2:** Unadjusted and adjusted prevalence rates (PR) and respective 95% confidence intervals (95%CI) of the COVID-19 vaccination schedule according to independent variables. Rio Grande, 2023 (n=1,925)

Variables	Unadjusted PR (95%CI)	p-value	Adjusted PR (95%CI)	p-value
Sex		0.002		0.003
Male	1.00		1.00	
Female	1.07 (1.03;1.12)		1.07 (1.02;1.12)	
Age group (years)		<0.001		<0.001
Up to 29	1.00		1.00	
30-39	1.11 (1.02;1.22)		1.12 (1.02;1.23)	
40-49	1.20 (1.10;1.31)		1.22 (1.12;1.33)	
50-59	1.26 (1.16;1.37)		1.28 (1.22;1.43)	
60 or over	1.32 (1.21;1.42)		1.32 (1.22;1.43)	
Marital status		0.251		0.064
Has a partner	1.00		1.00	
Does not have a partner	1.02 (0.98;1.07)		1.04 (1.00;1.09)	
Race/skin color		0.227		0.274
White	1.00		1.00	
Black	1.00 (0.94;1.07)		1.01 (0.94;1.07)	
Mixed-race/Asian/Indigenous	0.94 (0.87;1.01)		0.95 (0.88;1.02)	
Economic level		0.271		0.604
A/B (wealthiest)	1.00		1.00	
C	0.98 (0.94;1.02)		1.00 (0.96;1.04)	
D/E (poorest)	0.96 (0.87;1.06)		0.96 (0.87;1.06)	
Tobacco smoking		0.036		0.133
No	1.00		1.00	
Yes	0.93 (0.85;1.01)		0.92 (0.84;1.00)	
Former smoker	1.05 (1.01;1.11)		1.00 (0.95;1.05)	
Multimorbidity		<0.001		0.003
No	1.00		1.00	
Yes	1.13 (1.08;1.17)		1.07 (1.02;1.11)	
Guidance on importance of vaccination		0.011		0.007
No	1.00		1.00	
Yes	1.06 (1.01;1.11)		1.06 (1.02;1.11)	

**Figure 1:**
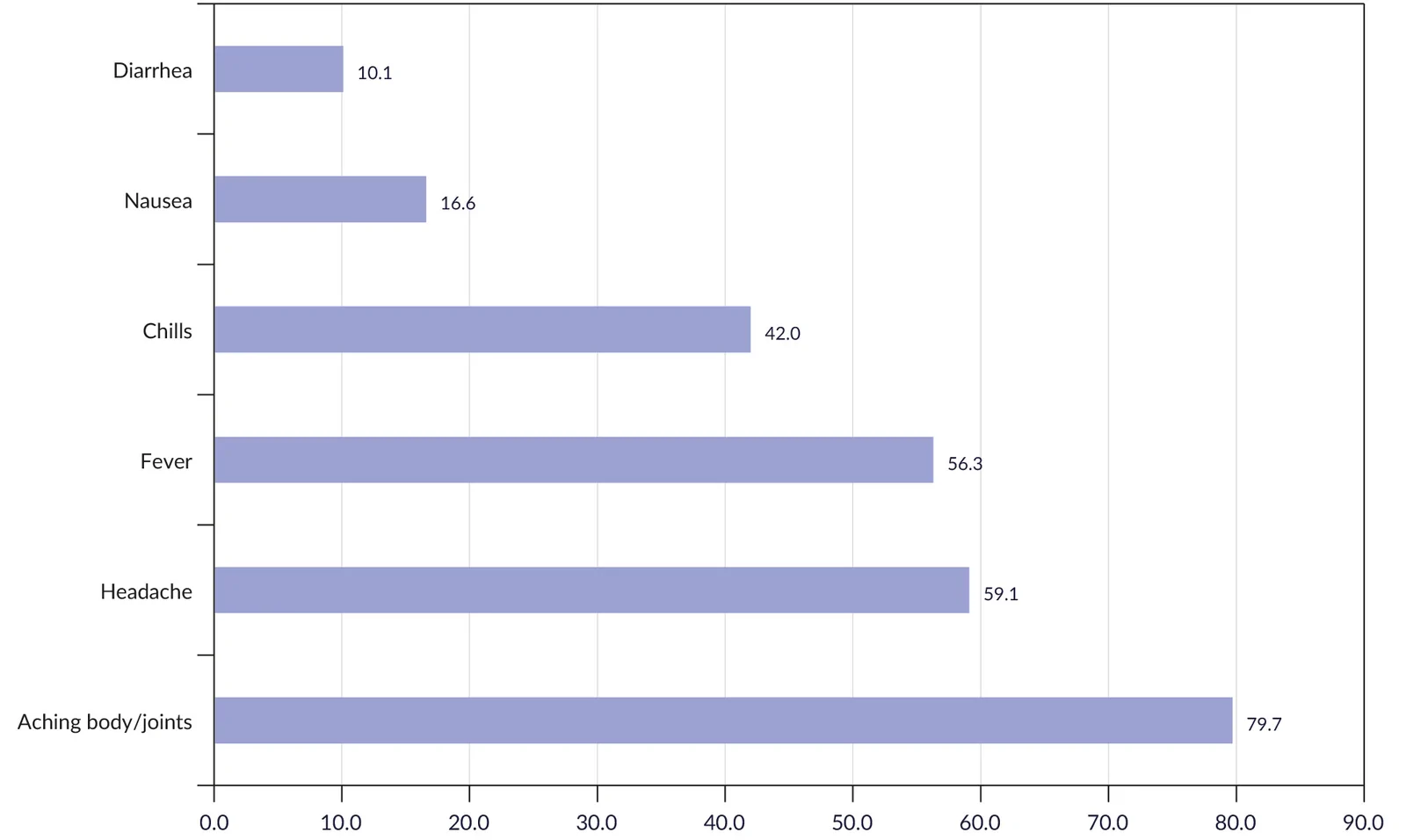
Prevalence of post-vaccination side effects most reported by interviewees who were vaccinated against COVID-19. Rio Grande, 2023 (n=1,046)

**Figure 2:**
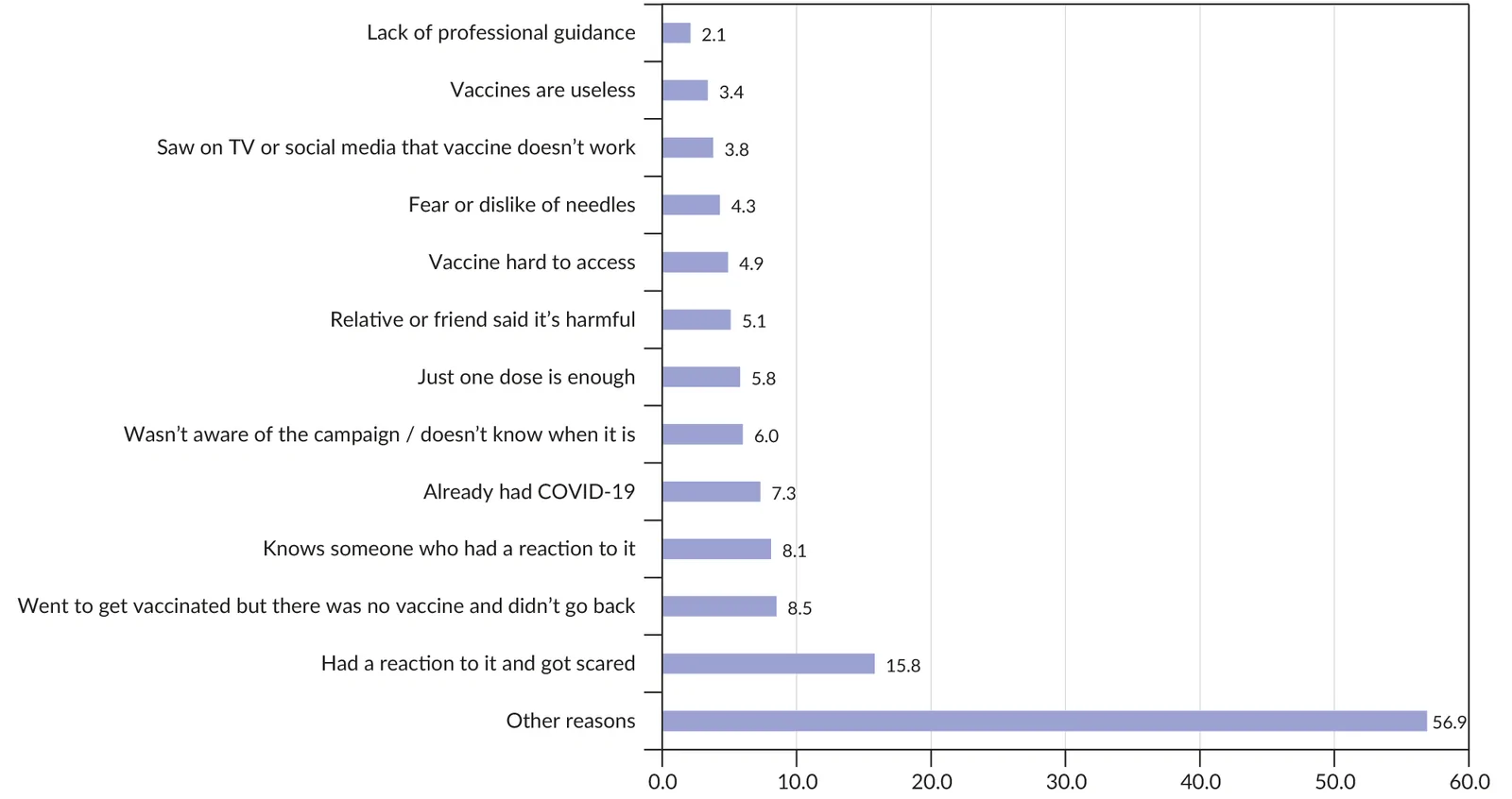
Prevalence of reasons for not getting vaccinated against COVID-19. Rio Grande, 2023 (n=304)

When asked about the reasons for not receiving any of the vaccine doses, 15.8% reported that they had a reaction and were afraid to take it again; 8.5% reported that when they went to get vaccinated, there was no vaccine available and they did not go back to get vaccinated; 8.1% knew someone who had a reaction and were afraid; 7.3% said they did not get vaccinated because they had already had COVID-19 (Figure 2).

## Discussion

The findings of this study reveal that vaccination adherence was higher among women, the elderly, those with more than one comorbidity, as well as those who received guidance on the importance of vaccination. Eight out of ten received three doses against COVID-19. However, these data should be interpreted with caution given some limitations that should be pointed out, such as the short time between vaccine having been made available and data collection, and differences in the number of vaccine doses offered by the government for each age group. Another limitation is that the data collection period coincided with the time when the WHO still considered COVID-19 to be an international public health emergency, which may have influenced the decision to get vaccinated. Another problem encountered in the data analysis was the operationalization of the race/skin color variable; it was necessary to combine the mixed-race, Asian and Indigenous categories to enable statistical testing. No statistical difference was found between the categories according to race/skin color; however, it is recommended that studies with representative samples of all racial characteristics of the Brazilian population be conducted in order to better assess possible differences in vaccination coverage.

In this study, women were vaccinated more than men. In the city of Porto Alegre, the total number of doses administered to women was 30% higher than those administered to men. In some age ranges, the percentage of women with a complete vaccination schedule was more than 55% higher than that of men ^5^. Considering all doses administered, the percentage of women who received the vaccine totaled 57%. Analyzing the state of Rio Grande do Sul in 2023, similar data are observed ^9^. Nationally, during the 2021-2022 COVID-19 vaccination campaign, adult women also showed higher vaccination coverage rates than men, ranging from 25% to 118% ^14^.

The fact that women are more likely to get vaccinated can be explained by the fact that, historically, they generally use health services more frequently. Men predominantly seek health services in emergency situations, for specialized or urgent health problems, and those who already have a diagnosis of morbidity are 49% more likely to seek the service compared to those without morbidity ^15^. In this context, men may be missing opportunities to receive guidance on the importance of vaccination and even the completeness of care, resulting in lower vaccine adherence.

This study showed that older people were more likely to get vaccinated. Data from E-SUS Rio Grande do Sul 2023 also demonstrated that older people between 60 and over 80 years old adhered more to the complete vaccination schedule ^9^. In the far north of Mato Grosso state, it was shown that most older people showed acceptance and desire to be immunized. Among the justifications, they pointed to reduction in mortality and protection of the population against the virus and severe forms of the disease ^16^. In Brazil, during the first two years of vaccination, it was shown that the vaccination coverage of the booster dose in December 2022 was 80%, 53% and 25% among older people, adults and adolescents, respectively ^14^. In Rio Grande do Sul state, in Palmeira das Missões, the profile of complete vaccination was that of older individuals and females, with the population that was least vaccinated being in the 20 to 29 age group ^17^.

The analysis of our study data showed that people with multimorbidity, that is, two or more concomitant health problems, had a 7% higher probability of vaccination when compared to those with no or only one disease (Table 2). In Bosnia and Herzegovina, in 2021, a higher proportion (25%) of vaccine administration was observed in individuals who received guidance on vaccination. Furthermore, 30% of respondents reported that they got vaccinated to protect their health ^18^. In a literature review on influenza vaccination, conducted between 2010 and 2020, people with comorbidities who participated in groups at health centers and, consequently, frequented services more often were more exposed to receiving educational guidance on immunization ^19^.

In our study, those who received guidance on vaccination were 6% more likely to get vaccinated. In Brazil, it was found that in 2021 and 2022, municipalities with the poorest health service indicators were more likely to have lower COVID-19 vaccination coverage, for example, the number of nurses and doctors per inhabitant, whereby in municipalities with fewer of these professionals the probability of having low coverage in adults was 2.40 times greater when compared to municipalities with a higher number of health professionals. Among the challenges to vaccine access are limited access to healthcare professionals and reliable sources of information, and low availability and quality of healthcare services ^14^. Lack of knowledge about the vaccine can negatively impact the decision to get vaccinated and indicates an urgent need to inform the population about the effectiveness and importance of COVID-19 vaccination, since this measure is for prevention and containment of transmission ^1^.

In this study, 41% of people who received the COVID-19 vaccine experienced side effects, the most commonly reported being body and/or joint pain, headache, fever and chills (Figure 1). In Mongolia, in 2021, the main reason for vaccine hesitancy among the general public was fear of possible side effects from a vaccine (29%). Common side effects include: headache, fever, injection site pain, muscle pain and fatigue ^20^. COVID-19 vaccines can cause side effects, although they are generally mild and short-lived. The most commonly reported are pain, redness or swelling at the injection site, fatigue or tiredness, headache, muscle or joint pain, chills, fever and nausea. These side effects usually occur in the first few days after receiving the vaccine and last only a few days ^21^.

In the analysis of the data of our study, the most frequently reported reasons for not completing the vaccination schedule were: experiencing a reaction and being afraid to get vaccinated again; when they went to get vaccinated, the vaccine was unavailable and they didn't return to get vaccinated; knowing someone who had a reaction and being afraid, as well as not getting vaccinated because they had already had COVID-19 (Figure 2). Other reasons were also mentioned by 57% of the sample; however, it was not possible to specify each one, as the study instrument did not allow this. Therefore, in order to delve deeper into the reasons, we suggest that qualitative studies, focused on the unvaccinated population, be conducted in order to identify existing gaps in quantitative methodologies.

Vaccine refusal has become a major challenge to achieving herd immunity in the quest to reduce COVID-19 transmission and, consequently, mortality caused by the coronavirus. Vaccine hesitancy is linked to several factors, such as vaccine side effects, misinformation about the need for vaccination, lack of trust in the health system, lack of knowledge about vaccines, disease severity, vaccine safety and efficacy, and whether the vaccine is provided free of charge by the government ^22^^,^^23^.

In the United States, in 2020, doubts about vaccines, lack of information about safety, fear of affecting reproductive health, laboratory preference, access, availability, and the spread of fake news were reported as causes that can be attributed to low vaccination uptake ^24^.

Implementation of COVID-19 vaccination in Brazil was notably slow, with high mortality rates due to facilitated transmission of the virus. Despite having a universal and comprehensive public health system, Brazil faced challenges such as differences in access to healthcare, the government's inadequate response to the pandemic, the rampant dissemination of anti-vaccine information, vaccination discouragement, and increasing vaccine hesitancy ^14^. The actions of the federal government in 2021-2022 were characterized by delays in purchases, denialism, conspiracy theories and skepticism regarding vaccines, creating a scenario of intentional disarticulation in the vaccination campaign. Although some state governments attempted to carry out joint actions, their pandemic response strategies did not prioritize monitoring and combating inequalities ^14^.

Discussions about the various forms of organization of the system and the services provided have occupied Brazilian Unified Health System (Sistema Único de Saúde, SUS) assistance policies. In general, the SUS has, at some periods, faced an inability to provide care due to a lack of funds, the urgent need to expand the number of beds and train teams, in addition to facing a crisis in the management of resources and supplies in order to guarantee comprehensive care. After a late and difficult start, mainly due to the fact that high-level health managers did not recognize the importance of vaccination as a form of prevention, vaccines were acquired and distributed, enabling vaccination of a large part of the population ^25^.

In 2023, the Ministry of Health launched a national movement for COVID-19 vaccination, emphasizing its importance and the value and leading role of the SUS in the adherence of the Brazilian population to the vaccine, as a pro-life movement ^26^. However, since January 2024, the vaccination schedule provided by the government is only aimed at priority groups, with one or two annual doses depending on the group to which the individual belongs. This may result in a drop in immunization among those who traditionally get vaccinated less, that is, men, young people and people without health problems.

This study demonstrated that the individuals with the highest vaccination rates in Rio Grande, Rio Grande do Sul state, were those who culturally frequent health services more often, such as women, the elderly and those who receive care for multimorbidity and, consequently, are more exposed to receiving guidance on vaccination. This highlights the need for campaigns that demonstrate the importance of men and younger, healthier people also protecting themselves through vaccination against a disease the long-term consequences of which are not yet fully understood and which has led to the deaths of millions of people worldwide.

## Data Availability

The data and analyses related to this research are not open access; however, they may be made available at any time upon request from the journal.
