# Supplementary material for: COVID-19 vaccination schedule, associated factors, and reasons for refusal in adults and the elderly: a population-based cross-sectional study, Rio Grande, Brazil, 2023
Source: Epidemiol Serv Saude. 2026 Mar 10;35:e20250503. doi: 10.1590/S2237-96222026v35e20250503.en (PMC13686317; doi:10.1590/S2237-96222026v35e20250503.en)
Supplement: Translation

# Translation: Esquema vacinal contra covid-19, fatores associados e motivos de recusa em adultos e idosos: estudo transversal de base populacional, Rio Grande, 2023

**DOI:** 10.1590/S2237-96222026v35e20250503.pt

**Authors:** Aline Augusta Medeiros Rutz, Mirelle de Oliveira Saes, Rosália Garcia Neves, Thales Rodrigues Almeida, Renata Cristina da Silva Martins, Suele Manjourany Silva Duro

**Affiliations:** 1 Universidade Federal de Pelotas, Programa de Pós-Graduação em Enfermagem, Pelotas, RS, Brasil Universidade Federal de Pelotas Programa de Pós-Graduação em Enfermagem Pelotas RS Brasil; 2 Universidade Federal do Rio Grande, Programa de Pós-Graduação em Ciências da Saúde, Rio Grande, RS, Brasil Universidade Federal do Rio Grande Programa de Pós-Graduação em Ciências da Saúde Rio Grande RS Brasil

**Keywords:** Cobertura Vacinal, Esquemas de Imunização, Vacinação, COVID-19, Estudos Transversais

## Abstract

**Objetivo:**

Estimar a prevalência da realização do esquema vacinal contra a covid-19 e investigar fatores associados e motivos de recusa em adultos e idosos residentes em Rio Grande, Rio Grande do Sul.

**Métodos:**

Estudo transversal, com participantes do Monitoramento de Indicadores de Saúde em Adultos e Idosos após Infecção pela covid-19 realizado em 2022-2023. O desfecho foi o esquema vacinal contra covid-19. Considerou-se o recebimento de pelo menos três doses da vacina. As variáveis de exposição foram características sociodemográficas e de morbidades. Também foram investigados os motivos da não realização do esquema vacinal. Foram calculadas as prevalências e razões de prevalências brutas e ajustadas.

**Resultados:**

Dos 1.925 entrevistados, 83,3% receberam as três doses da vacina. A prevalência de realização foi maior entre mulheres (85,5%), idosos (92,6%), respondentes que nunca fumaram (83,3%) ou ex-fumantes (87,6%), com multimorbidade (88,6%) e que foram orientados sobre a importância da vacina (85,1%). Daqueles que não completaram o esquema, 15,8% não o fizeram porque tiveram reação; 8,5% quando foram tomar não tinham o imunobiológico disponível e não voltaram a procurar; 8,1% conheciam alguém que teve reação e ficaram receosos e 7,3% alegaram que tiveram covid-19.

**Conclusão:**

Um total de 83,3% realizaram o esquema vacinal de pelo menos três doses contra covid-19. A adesão à vacinação deve ser incentivada especialmente nos grupos que apresentaram menores coberturas, a exemplo dos homens, pessoas mais jovens e sem morbidade.

**Aspectos éticos**

Esta pesquisa respeitou os princípios éticos, obtendo os seguintes dados de aprovação:

Comitê de ética em pesquisa: Universidade Federal do Rio Grande

Número do parecer: 4.375.697

Data de aprovação: 3/11/2020

Certificado de apresentação de aprecia-ção ética: 39081120.0.0000.5324

Registro do consentimento livre e escla-recido: Os princípios éticos foram garantidos por meio da leitura do Termo de Consentimento Livre e Esclarecido, respeitando o direito de não participação na pesquisa e do anonimato dos sujeitos.

## Introduction

A covid-19 foi identificada em dezembro de 2019 na China. Devido à sua alta transmissibilidade, a Organização Mundial de Saúde (OMS) declarou estado de emergência de saúde pública de alcance internacional, orientando os países a adotarem medidas preventivas, como isolamento social e o uso de máscaras. As vacinas surgiram como uma ferramenta importante para a prevenção dessa doença infecciosa ^1^^,^^2^.

Em maio de 2023, o diretor-geral da OMS declarou o fim da emergência de saúde pública relativa à covid-19. Apesar de seguir a recomendação do Comitê de Emergências da OMS, o diretor alertou que a covid-19 continuava sendo uma ameaça à saúde mundial. Portanto, os países, seus sistemas de saúde e suas populações deveriam continuar adotando as medidas de prevenção. Entre as principais recomendações consideradas, destacou-se a vacina ^3^.

O Ministério da Saúde, em novembro de 2022, reforçou que os brasileiros precisavam tomar as doses de reforço contra o coronavírus para uma efetiva proteção. Entretanto, nesse mesmo ano, mais de 80 milhões de pessoas deixaram de comparecer aos postos de vacinação para receber a primeira dose de reforço. A estratégia de reforçar o calendário vacinal aumentou em mais de cinco vezes a proteção contra casos graves e óbitos pela covid-19. No Brasil, até setembro de 2023, apenas 16% da população foi imunizada com a vacina bivalente ^4^^,^^5^^,^^6^.

Há importantes disparidades no percentual de realização da vacinação em diferentes países, variando entre 5% no Haiti e 100% nos Emirados Árabes Unidos ^6^. Entre os motivos de recusa, destacam-se medo dos efeitos colaterais, desconfiança nas políticas públicas, a origem das vacinas, não acreditar na eficácia e a falta de segurança nas vacinas. Outros motivos de destaque são: já ter se infectado com o vírus, falta de dados clínicos sobre a vacina, acreditar que a covid-19 era uma doença inofensiva, acreditar em teorias de conspiração e não ser do grupo de risco para agravamento da doença ^7^^,^^8^.

Este estudo justifica-se pela importância de identificar a adesão à vacinação contra a covid-19, traçando o perfil daqueles que menos se vacinaram. Acredita-se que, assim, políticas públicas possam ser propostas e desenvolvidas objetivando a ampliação das coberturas vacinais na população.

O objetivo deste estudo foi estimar a prevalência da realização do esquema vacinal contra a covid-19 e investigar fatores associados e motivos de recusa em adultos e idosos residentes em Rio Grande, localizada na região Sul do Brasil.

## Métodos

Delineamento

Trata-se de um estudo transversal aninhado a uma coorte. Foram utilizados dados da segunda coleta do estudo longitudinal denominado Monitoramento de Indicadores de Saúde em Adultos e Idosos após Infecção pela covid-19 Residentes em Rio Grande, Rio Grande do Sul.

Participantes e contexto

A pesquisa foi realizada em Rio Grande, Rio Grande do Sul. Situado no extremo sul do estado, o município conta com uma população estimada de 210 mil habitantes. Até janeiro de 2024, teve cerca de 60.834 casos confirmados e uma taxa de mortalidade de aproximadamente 346.4 por 100 mil habitantes ^9^.

No estudo de linha de base, a amostra incluiu indivíduos com 18 anos ou mais que tiveram diagnóstico de covid-19 confirmado por meio do teste Reação em Cadeia da Polimerase com Transcrição Reversa (Reverse Transcription Polymerase Chain Reaction, RT-PCR) entre dezembro de 2020 e março de 2021; que apresentaram sintomas durante a infecção; e que residiam em Rio Grande. Excluíram-se indivíduos com limitações funcionais e/ou doenças neurológicas avançadas que não pudessem responder ao questionário e aqueles privados de liberdade (prisões ou instituições de longa permanência para idosos). A coleta de dados foi realizada em duas etapas consecutivas: 1) Coleta telefônica: Foram realizados até cinco contatos telefônicos com cada indivíduo elegível para o estudo. Estes contatos eram feitos em dias e horários alternados. Após cinco contatos sem sucesso, era enviada mensagem padronizada aos telefones com WhatsApp. Com os indivíduos que respondiam a mensagem enviada, era agendada entrevista por telefone ou presencial, de acordo com a preferência do respondente. Aqueles que não atendiam ao telefone e não respondiam à mensagem eram alocados para a etapa seguinte, de coleta domiciliar. 2) Coleta domiciliar: Nesta etapa do estudo o entrevistador identificado ia até o domicílio do entrevistado realizar a entrevista com aqueles que não foram localizados durante a etapa de coleta telefônica. O instrumento e método de aplicação foram os mesmos da coleta telefônica. Consideraram-se perdas indivíduos que não foram localizados após cinco tentativas de contato telefônico, uma via WhatsApp e três visitas domiciliares.

Para localizar a amostra, no estudo de linha de base, foi realizado contato com a Secretaria Municipal de Saúde de Rio Grande e obteve-se a listagem de todas as notificações realizadas. Foram selecionados indivíduos sintomáticos, com resultado positivo a partir de teste RT-PCR e que tivessem fornecido dados completos (nome, endereço, telefone e presença de sintomas). O total foi de 3.822 elegíveis para o estudo; destes, foram entrevistados 2.919 indivíduos nessa etapa ^10^.

Para o estudo de acompanhamento, foram contatados os 2.919 indivíduos entrevistados na linha de base. A coleta de dados ocorreu em duas etapas: 1) entrevistas telefônicas e 2) entrevistas domiciliares, conforme ocorreu durante o estudo de linha de base. Deu-se início por meio de ligações telefônicas conduzidas por entrevistadores previamente treinados. Quando possível, a entrevista era realizada logo no primeiro contato telefônico, ou, se necessário, agendados dia e horário. Para os indivíduos que não atenderam a cinco ligações, tentou-se contato via Whatsapp. Além das entrevistas telefônicas, foi realizada visita domiciliar para a coleta dos dados daqueles com receio de responder ao contato telefônico e para aqueles que não atenderam a nenhuma das cinco tentativas telefônicas.

A coleta de dados foi realizada entre dezembro de 2022 e maio de 2023 o instrumento continha dados gerais; perguntas sobre sintomas remanescentes da infecção pela covid-19; suporte social e recebimento de orientações; hábitos de vida; comportamentos; saúde; uso de serviços de saúde; vacinação; e dados socioeconômicos.

Variáveis

O desfecho do estudo foi medido a partir da questão "Quantas doses da vacina contra a covid-19 o(a) Sr.(a) tomou?". A variável foi categorizada em 0-2 e 3-4 doses (número máximo de doses disponíveis na data da coleta de dados). Foram considerados vacinados os indivíduos que tomaram pelo menos três doses.

As variáveis independentes utilizadas foram: sexo (masculino; feminino); faixa etária em anos (até 29 anos; 30-39; 40-49; 50-59; e 60 anos ou mais); nível econômico, conforme Critério Brasil proposto pela Associação Brasileira de Empresas de Pesquisa (ABEP) (A/B; C; D/E), em que A é a categoria dos mais ricos e a D/E, dos mais pobres ^11^; raça/cor da pele (branca; preta; parda/amarela/indígena); situação conjugal (com companheiro; sem companheiro); tabagismo (não; sim; ex-fumante); multimorbidade (não; sim); e recebimento de orientação sobre a importância da vacina (não; sim). Para a variável raça/cor da pele, houve coleta nas cinco categorias, mas para fins de análise estatística, juntou-se parda, amarela e indígena em uma só. Considera-se multimorbidade quando o indivíduo tem dois ou mais problemas concomitantes da seguinte lista: doença mental ou emocional; problemas respiratórios; osteoporose ou problema articular; hipertensão; diabetes; problemas cardíacos; problemas nos olhos; câncer; doenças neurológicas.

Para avaliar os motivos de não-realização do esquema vacinal, perguntou-se: "O(A) Sr.(a) está com o esquema vacinal completo de acordo com sua idade?"(não;sim). Nesse caso, considera-se esquema vacinal completo quando o indivíduo tomou pelo menos três doses da vacina contra covid-19. Em caso de a resposta ser "não", na sequência investigou-se o motivo da seguinte forma: "Vou ler alguns motivos e o(a) Sr.(a) me informa qual(is) o(s) mais próximo(s) da sua realidade. Os motivos são: 1) já tive covid-19; 2) tive reação e fiquei com medo; 3) medo/não gosta de agulha; 4) parente/amigo falou que faz mal; 5) conhece alguém que teve reação; 6) falta de orientação profissional; 7) dificuldade de acesso à vacina; 8) vacinas não servem para nada; 9) uma dose já é o suficiente; 10) não ficou sabendo/não sabe quando é a campanha; 11) quando fui tomar não tinha e não voltei a procurar; 12) vi na TV ou rede social que a vacina não funciona ou faz mal".

Métodos estatísticos

Foi realizada análise descritiva com obtenção das proporções, análise bivariada do desfecho conforme variáveis independentes por meio do teste qui-quadrado para heterogeneidade e tendência linear. As análises bruta e ajustada foram conduzidas por meio de regressão de Poisson, com estimativa robusta da variância, segundo modelo hierárquico. Variáveis com p-valor≤0,20 foram mantidas no modelo para controle de possíveis fatores de confusão. O modelo hierárquico foi composto por três níveis assim distribuídos: 1) sexo, idade, nível econômico, raça/cor da pele e situação conjugal; 2) tabagismo; 3) multimorbidade e recebimento de orientações sobre a importância da vacinação contra a covid-19. A análise dos dados foi realizada utilizando o programa Stata, versão 15.0 (Stata Corporation, College Station, Texas, USA) e foi considerado um nível de significância de 5%.

## Resultados 

Foram entrevistados 1.925 indivíduos no estudo de acompanhamento, o que totalizou 65,9% da amostra inicial de 2.919 indivíduos. Destes, 1.179 eram mulheres (61,3%), 1.197 tinham até 49 anos (62,2%), 1.148 relataram ter companheiro (59,9%), 1.486 se autodeclararam de raça/cor da pele branca (77,6%) e 1.499 nunca tinham fumado (78,9%). Além disso, 941 estavam no nível econômico C (54,1%), 855 tinham multimorbidade (46,3%), 1.153 receberam orientações sobre a importância da vacinação contra a covid-19 (60, 4%) e 1.046 relataram ter tido algum efeito colateral após a vacinação (58,6%) (Tabela 1).

A prevalência de recebimento de três doses ou mais da referida vacina foi de 83,3% (n=1.511), sendo estatisticamente mais frequente entre as mulheres, entre os indivíduos mais velhos, entre os ex-fumantes e aqueles que nunca fumaram, em pessoas com multimorbidade e que receberam orientações sobre a importância da vacinação. Não foram encontradas diferenças estatísticas na prevalência de vacinação conforme a situação conjugal, raça/cor da pele e nível econômico (Tabela 1).

Após análise ajustada, foi evidenciado que as mulheres apresentaram probabilidade 7% maior do que os homens (RP 1,07; IC95% 1,02; 1,12) de receber a vacina. Houve relação direta entre a probabilidade de receber a vacina conforme o aumento da idade; entre aqueles com 60 anos ou mais foi de 1,32 vezes maior do que entre os indivíduos de até 29 anos (RP 1,32; IC95% 1,22; 1,43). Pessoas com multimorbidade (RP 1,07; IC95% 1,02; 1,11) e que receberam orientações sobre a importância da vacinação (RP 1,06; IC95% 1,02; 1,11) também tiveram maior probabilidade de se vacinar (Tabela 2).

Quanto aos efeitos colaterais, os mais relatados foram dor no corpo e/ou articulares (79,7%), dor de cabeça (59,1%), febre (56,3%) e calafrios (42,0%) (Figura 1).

Quando questionados sobre os motivos de não realização de alguma das doses da vacina, 15,8% informaram que tiveram reação e ficaram com medo de tomar de novo; 8,5% relataram que, quando foram tomar, não tinha vacina e não voltaram a procurar; 8,1% conheciam alguém que teve reação e ficaram com medo; 7,3% disseram que não tomaram porque já tiveram covid-19 (Figura 2).

**Tabela 1:** Distribuição da amostra e prevalência (%) do esquema vacinal contra a covid-19 de acordo com variáveis independentes. Rio Grande, 2023 (n=1.925)

Variáveis	Amostra (%)	Esquema vacinal (%)	p-valor
Amostra total		1.511 (83,3)	
Sexo			0,001
Masculino	746 (38,8)	554 (79,6)	
Feminino	1.179 (61,3)	955 (85,5)	
Faixa etária (anos)			<0,001
Até 29	311 (16,2)	207 (70,4)	
30-39	442 (23,0)	331 (78,4)	
40-49	444 (23,1)	352 (84,6)	
50-59	339 (17,6)	280 (88,9)	
60 ou mais	389 (20,2)	339 (92,6)	
Situação conjugal			0,256
Com companheiro	1.148 (59,9)	889 (82,5)	
Sem companheiro	770 (40,2)	616 (84,5)	
Raça/cor da pele			0,160
Branca	1.486 (77,6)	1.172 (83,8)	
Preta	198 (10,3)	158 (84,0)	
Parda/amarela/indígena	230 (12,0)	169 (78,6)	
Nível econômico			0,538
A/B (mais ricos)	701 (40,3)	592 (84,7)	
C	941 (54,1)	779 (82,9)	
D/E (mais pobres)	98 (5,6)	80 (81,6)	
Tabagismo			0,033
Não	1.499 (78,9)	1.185 (83,3)	
Sim	166 (8,7)	124 (77,5)	
Ex-fumante	236 (12,4)	197 (87,6)	
Multimorbidade			<0,001
Não	992 (53,7)	748 (78,7)	
Sim	855 (46,3)	733 (88,6)	
Orientações sobre importância da vacina			0,009
Não	756 (39,6)	575 (80,4)	
Sim	1.153 (60,4)	931 (85,1)	
Doses de vacina contra a covid-19			
0 a 2 doses	304 (16,8)	-	
3 ou 4 doses	1.511 (83,3)	-	
Efeitos colaterais pós-vacina			
Não	739 (41,4)	-	
Sim	1.046 (58,6)	-	

**Tabela 2:** Razões de prevalência (RP) bruta e ajustada e respectivos intervalos de confiança de 95% (IC95%) do esquema vacinal contra a covid-19 de acordo com variáveis independentes. Rio Grande, 2023 (n=1.925)

Variáveis	RP bruta (IC95%)	p-valor	RP ajustada (IC95%)	p-valor
Sexo		0,002		0,003
Masculino	1,00		1,00	
Feminino	1,07 (1,03;1,12)		1,07 (1,02;1,12)	
Faixa etária (anos)		<0,001		<0,001
Até 29	1,00		1,00	
30-39	1,11 (1,02;1,22)		1,12 (1,02;1,23)	
40-49	1,20 (1,10;1,31)		1,22 (1,12;1,33)	
50-59	1,26 (1,16;1,37)		1,28 (1,22;1,43)	
60 ou mais	1,32 (1,21;1,42)		1,32 (1,22;1,43)	
Situação conjugal		0,251		0,064
Com companheiro	1,00		1,00	
Sem companheiro	1,02 (0,98;1,07)		1,04 (1,00;1,09)	
Raça/cor da pele		0,227		0,274
Branca	1,00		1,00	
Preta	1,00 (0,94;1,07)		1,01 (0,94;1,07)	
Parda/amarela/indígena	0,94 (0,87;1,01)		0,95 (0,88;1,02)	
Nível econômico		0,271		0,604
A/B (mais ricos)	1,00		1,00	
C	0,98 (0,94;1,02)		1,00 (0,96;1,04)	
D/E (mais pobres)	0,96 (0,87;1,06)		0,96 (0,87;1,06)	
Tabagismo		0,036		0,133
Não	1,00		1,00	
Sim	0,93 (0,85;1,01)		0,92 (0,84;1,00)	
Ex-fumante	1,05 (1,01;1,11)		1,00 (0,95;1,05)	
Multimorbidade		<0,001		0,003
Não	1,00		1,00	
Sim	1,13 (1,08;1,17)		1,07 (1,02;1,11)	
Orientações sobre a importância da vacina		0,011		0,007
Não	1,00		1,00	
Sim	1,06 (1,01;1,11)		1,06 (1,02;1,11)	

**Figura 1:** Prevalência dos efeitos colaterais pós-vacina mais relatados pelos entrevistados que receberam a vacina contra a covid-19. Rio Grande, 2023 (n=1.046)

**Figura 2:** Prevalência dos motivos de não realização da vacinação contra a covid-19. Rio Grande, 2023 (n=304)

## Discussão 

Os achados deste estudo revelam que a adesão à vacinação foi maior entre as mulheres, pessoas idosas, que apresentavam mais de uma morbidade e entre aquelas que receberam orientações sobre a importância da vacinação. Oito a cada dez receberam três doses contra covid-19. Entretanto, esses dados devem ser interpretados com parcimônia tendo em vista algumas limitações que merecem ser apontadas, a exemplo do curto espaço de tempo entre a oferta da vacina e a coleta de dados e diferenças entre o número de doses vacinais ofertadas pelo governo para cada faixa etária. Outra limitação é que o período de coleta compreendeu aquele em que a OMS ainda considerava a covid-19 uma emergência de saúde pública internacional, o que pode ter influenciado na decisão de tomar a vacina. Outro problema enfrentado na análise de dados foi a operacionalização da variável raça/cor da pele; foi necessário juntar as categorias parda, amarela e indígena para possibilitar o teste estatístico. Não foi encontrada diferença estatística entre as categorias de acordo com a raça/cor da pele, entretanto recomenda-se que estudos com amostras representativas de todas as características raciais da população brasileira sejam feitos com intuito de avaliar melhor as possíveis diferenças em relação à cobertura vacinal.

Neste estudo, as mulheres se vacinaram mais que os homens. Em Porto Alegre, o total de doses aplicadas em mulheres foi 30% maior do que as realizadas em homens. Em alguns intervalos de faixa etária, a porcentagem de mulheres com esquema completo chegou a ser 55% maior do que a de homens ^5^. Se consideradas todas as doses aplicadas, a porcentagem de mulheres que tomaram vacina somou 57%. Analisando o estado do Rio Grande do Sul, em 2023, dados similares são observados ^9^. Em nível nacional, durante a campanha de vacinação contra a covid-19 em 2021-2022, mulheres adultas também apresentaram taxas de cobertura vacinal superiores às dos homens, variando entre 25% e 118% ^14^.

O fato de as mulheres apresentarem maior probabilidade para se vacinar pode ser explicado porque, historicamente, elas frequentam mais os serviços de saúde de forma geral. Homens procuram serviços de saúde predominantemente em situações de emergência, agravos na saúde em nível especializado ou de urgência, sendo que aqueles que já tem o diagnóstico de morbidade apresentam 49% maior probabilidade de buscarem o serviço em relação aos que não tem morbidade ^15^. Nesse contexto, pode ser que os homens estejam perdendo oportunidade de receberem orientações sobre a importância da vacinação e até mesmo a completude do cuidado, fazendo com que a adesão à vacina seja menor.

Neste estudo, evidenciou-se que os mais velhos se vacinaram mais. Dados do E-SUS Rio Grande do Sul 2023 também demonstraram que idosos entre 60 e mais de 80 anos aderiram mais ao esquema de vacinação completo ^9^. No extremo norte de Mato Grosso foi evidenciado que a maioria dos idosos demonstrou aceitação e desejo em ser imunizado. Entre as justificativas, apontaram a redução da mortalidade e a proteção da população contra o vírus e formas graves da doença ^16^. No Brasil, durante os dois primeiros anos de vacinação, demonstrou-se que a cobertura vacinal da dose de reforço em dezembro de 2022 foi de 80%, 53% e 25% entre idosos, adultos e adolescentes, respectivamente ^14^. No Rio Grande do Sul, em Palmeira das Missões, o perfil de vacinação completa foi de indivíduos mais velhos e do sexo feminino, sendo a população que menos se vacinou na faixa etária de 20 a 29 anos ^17^.

No âmbito desta análise, observou-se que pessoas com multimorbidade, ou seja, dois ou mais problemas de saúde concomitantes, apresentaram uma probabilidade 7% maior de vacinação quando comparadas àquelas com nenhuma ou apenas uma doença (Tabela 2). Na Bósnia e Herzegovina, em 2021, evidenciou-se uma maior proporção de realização da vacina em indivíduos (25%) que receberam orientações sobre vacinação. Ainda, 30% dos entrevistados informaram que se vacinaram para proteger sua saúde ^18^. Em uma revisão de literatura sobre vacinação contra a influenza, realizada entre 2010 e 2020, pessoas que apresentavam comorbidades, participavam de grupos nas unidades de saúde e, consequentemente, frequentavam mais os serviços estavam mais expostas a receber orientações educacionais sobre a imunização ^19^.

No presente estudo, aqueles que receberam orientações sobre a vacinação apresentaram probabilidade 6% maior de se vacinar. No Brasil, evidenciou-se que nos anos 2021 e 2022, os municípios com piores indicadores de serviços de saúde tiveram maior probabilidade de menor cobertura vacinal contra a covid-19 no Brasil, a exemplo do número de enfermeiros e médicos por habitantes, sendo que os municípios com menor número desses profissionais apresentaram 2,40 maior probabilidade de ter cobertura baixa em adultos quando comparados aos municípios com maior número de profissionais de saúde. Entre os desafios para o acesso à vacina, o acesso limitado aos profissionais de saúde e fontes confiáveis de informações, e pouca disponibilidade e qualidade dos serviços de saúde ^14^. A falta de conhecimento sobre a vacina pode ter impacto negativo sobre a tomada de decisão de se vacinar e indica uma necessidade urgente de informar a população sobre a eficácia e a importância de se vacinar contra a covid-19, uma vez que essa medida é de prevenção e contingenciamento da transmissão ^1^.

Neste estudo, 41% das pessoas que receberam a vacina da covid-19 apresentaram efeitos colaterais, sendo os mais relatados dor no corpo e/ou articulares, dor de cabeça, febre e calafrios (Figura 1). Na Mongólia, em 2021, a principal razão para a hesitação vacinal entre o público em geral foi o medo dos possíveis efeitos colaterais de uma vacina (29%). Os comuns incluem: dor de cabeça, febre, dor no local da injeção, dor muscular e fadiga ^20^. As vacinas contra a covid-19 podem causar efeitos colaterais, embora sejam geralmente leves e de curta duração. Os mais comumente relatados são dor, vermelhidão ou edema no local da injeção, fadiga ou cansaço, dor de cabeça, dores musculares ou articulares, calafrios, febre e náusea. Esses efeitos colaterais geralmente ocorrem nos primeiros dias após receber a vacina e duram apenas alguns dias ^21^.

Nesta análise, os motivos mais relatados para não completar o esquema vacinal foram: apresentou reação e ficou com medo de tomar novamente, quando foi tomar não tinha e não voltou a procurar, conhece alguém que teve reação e ficou com medo e não tomou porque já teve covid-19 (Figura 2). Outros motivos também foram mencionados por 57% da amostra, todavia não foi possível especificar cada um deles, visto que o instrumento do estudo não permitia. Diante disso, para aprofundar-se mais acerca dos motivos, sugere-se que estudos qualitativos, voltados para a população que não se vacina, sejam feitos, a fim de identificar lacunas existentes nas metodologias quantitativas.

A recusa vacinal se tornou um grande desafio para alcançar a imunidade coletiva na busca pela diminuição da transmissão da covid-19 e, consequentemente, da mortalidade causada pelo coronavírus. A hesitação vacinal está ligada a alguns fatores, como os efeitos colaterais da vacina, desinformação sobre a necessidade de vacinação, falta de confiança no sistema de saúde, falta de conhecimento sobre as vacinas, gravidade da doença, a segurança e eficácia da vacina e se a vacina é fornecida gratuitamente pelo governo (22, 23).

Nos Estados Unidos, em 2020, as dúvidas sobre as vacinas, a falta de informações sobre a segurança, o medo de afetar a saúde reprodutiva, a preferência por laboratório, o acesso, a disponibilidade e a disseminação de notícias falsas foram relatados como causas que podem ser atribuídas à baixa adesão à vacinação ^24^.

A implementação da vacinação contra a covid-19 no Brasil foi notavelmente lenta, com altos índices de mortalidade devido à fácil transmissão do vírus. Apesar de ter um sistema de saúde público, universal e abrangente, o país enfrentou desafios, como as diferenças no acesso aos cuidados de saúde, a resposta inadequada do governo à pandemia, a disseminação desenfreada de informação antivacina, desincentivos à vacinação e crescente hesitação em vacinar ^14^. As ações do governo federal em 2021-2022 foram caracterizadas por atrasos nas compras, negacionismo, teorias da conspiração e ceticismo em relação às vacinas, criando um cenário de intencional desarticulação na campanha de vacinação. Embora alguns governos estaduais tenham tentado realizar ações conjuntas, suas estratégias de resposta à pandemia não priorizaram monitorar e combater as desigualdades ^14^.

Discussões sobre as diversas formas de organização do sistema e dos serviços prestados ocuparam as políticas de assistência no Sistema Único de Saúde (SUS). De forma geral, o SUS se viu, em alguns períodos, diante de uma incapacidade assistencial por falta de verbas, a necessidade emergencial da expansão da quantidade de leitos e capacitação das equipes, além do enfrentamento à uma crise de gestão de recursos e insumos de modo a garantir assistência integral. Após um início tardio e difícil, principalmente pelo fato de os gestores não reconhecerem a importância da vacinação como forma de prevenção, as vacinas foram adquiridas e distribuídas, possibilitando a vacinação de grande parte da população ^25^.

O Ministério da Saúde, em 2023, iniciou um movimento nacional pela vacinação contra a covid-19, enfatizando a sua importância e a valorização e o protagonismo do SUS na adesão da população brasileira à vacina, como um movimento pró-vida ^26^. No entanto, desde janeiro de 2024, o esquema vacinal disponibilizado pelo governo é apenas voltado a grupos prioritários, com uma ou duas doses anuais a depender do grupo ao qual o indivíduo pertença. Isso pode vir a resultar em queda na imunização entre aqueles que tradicionalmente menos se vacinam, isto é, homens, jovens e pessoas sem problemas de saúde.

Evidenciou-se que os indivíduos que tiveram maior adesão à vacinação em Rio Grande, Rio Grande do Sul, foram aqueles que culturalmente mais frequentam os serviços de saúde, como as mulheres, os idosos e os que fazem acompanhamento de sua multimorbidade e, consequentemente, estão mais expostos ao recebimento de orientação sobre a vacinação. Ressalta-se a necessidade de campanhas que demonstrem a importância de homens e pessoas mais jovens e saudáveis também se prevenirem por meio da vacinação contra uma doença que ainda não tem todas suas sequelas tão bem conhecidas e que levou milhões de pessoas ao redor do mundo à morte.

## Data Availability

Os dados e as análises referentes à pesquisa não são de acesso aberto, entretanto poderão ser disponibilizados a qualquer momento mediante solicitação da revista.
